# In-situ Coronary Thrombosis in Antiphospholipid Syndrome: A Case Report

**DOI:** 10.7759/cureus.5727

**Published:** 2019-09-23

**Authors:** Hira Pervez, Sharan Rufus, Prabhakaran Gopalakrishnan

**Affiliations:** 1 Internal Medicine / Cardiology, Dow University of Health Sciences (DUHS), Karachi, PAK; 2 Cardiology, Canton Medical Education Foundation, Canton, USA; 3 Cardiology, Northeast Ohio Medical University / Aultman Hospital, Canton, USA

**Keywords:** coronary in-situ thrombus, anti-phospholipid syndrome, coronary thrombosis in systemic diseases, aps, thrombosis, coronary arteries

## Abstract

Antiphospholipid antibody syndrome (APS) is a systemic autoimmune disorder characterized by arterial and venous thrombosis, often accompanied by elevated titers of anti-phospholipid antibodies. Cardiac involvement in APS is not uncommon. However, acute myocardial infarction (AMI) from in-situ thrombosis is a rare but important manifestation of APS. We present a rare case of AMI in a young female with APS secondary to in-situ coronary thrombosis.

## Introduction

Coronary artery disease presenting with acute myocardial infarction (AMI) may be the first manifestation of antiphospholipid antibody syndrome (APS), most commonly seen in young females. Mechanisms of ischemia can include accelerated atherosclerosis, coronary thromboembolism, or in-situ thrombus formation in coronary arteries [1-2]. Herein, we present a rare case of AMI in a young female with APS secondary to in-situ coronary thrombosis. We discuss the potential mechanisms of myocardial ischemia in APS and underscore the importance of recognizing this clinical presentation, as timely diagnosis may be life-saving.

## Case presentation

**Figure 1:**
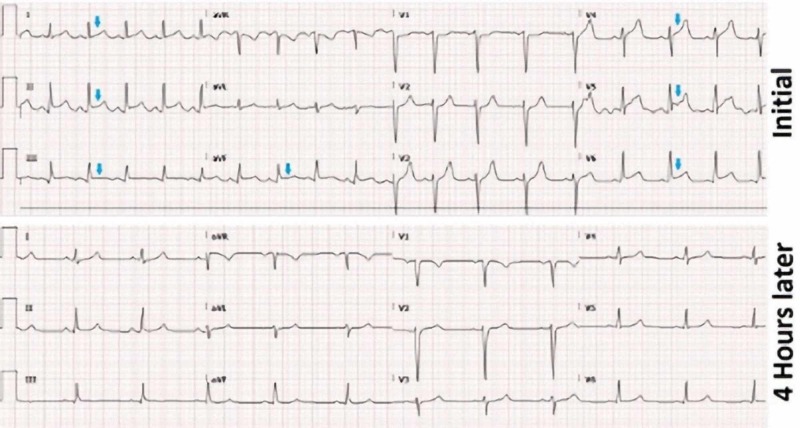
Electrocardiogram. ST-segment elevation seen on presentation (arrow, above); ST-segment elevation resolved four hours later (below)

A 27-year-old female with a past medical history of chronic pain syndrome presented to the emergency department (ED) with complaints of generalized body fatigue and pain, with severe retrosternal chest pain over four hours duration, which initially worsened on lying down and improved with sitting up. On her presentation in the ED, the heart rate was 89 beats per minute, blood pressure was 128/80 mm Hg, and oxygen saturation was 100% on room air. Initial electrocardiogram (EKG) showed diffuse ST-segment elevations in anterior (V4 through V6) and inferior leads (II, III, aVF) (Figure 1). She presented in the past with similar symptoms and was under management by the pain clinic for chronic pain syndrome.

Routine investigations were sent and serial troponin levels were measured. The first troponin was 0.023 (normal high 0.040 ng/ml). An emergent echocardiogram (ECHO) was done, which showed a preserved ejection fraction with no regional wall motion abnormalities. A repeat EKG after 15 minutes showed persistent ST elevation without any evolutionary changes typical of ST-elevation myocardial infarction (STEMI). This presentation with atypical chest pain, negative cardiac biomarkers, and normal ECHO was more compatible with acute pericarditis rather than acute coronary syndrome (ACS). The patient was admitted for close monitoring and further workup. Empiric treatment for possible pericarditis was started and the patient was followed by serial EKGs and cardiac biomarkers while awaiting the lab work.

**Figure 2:**
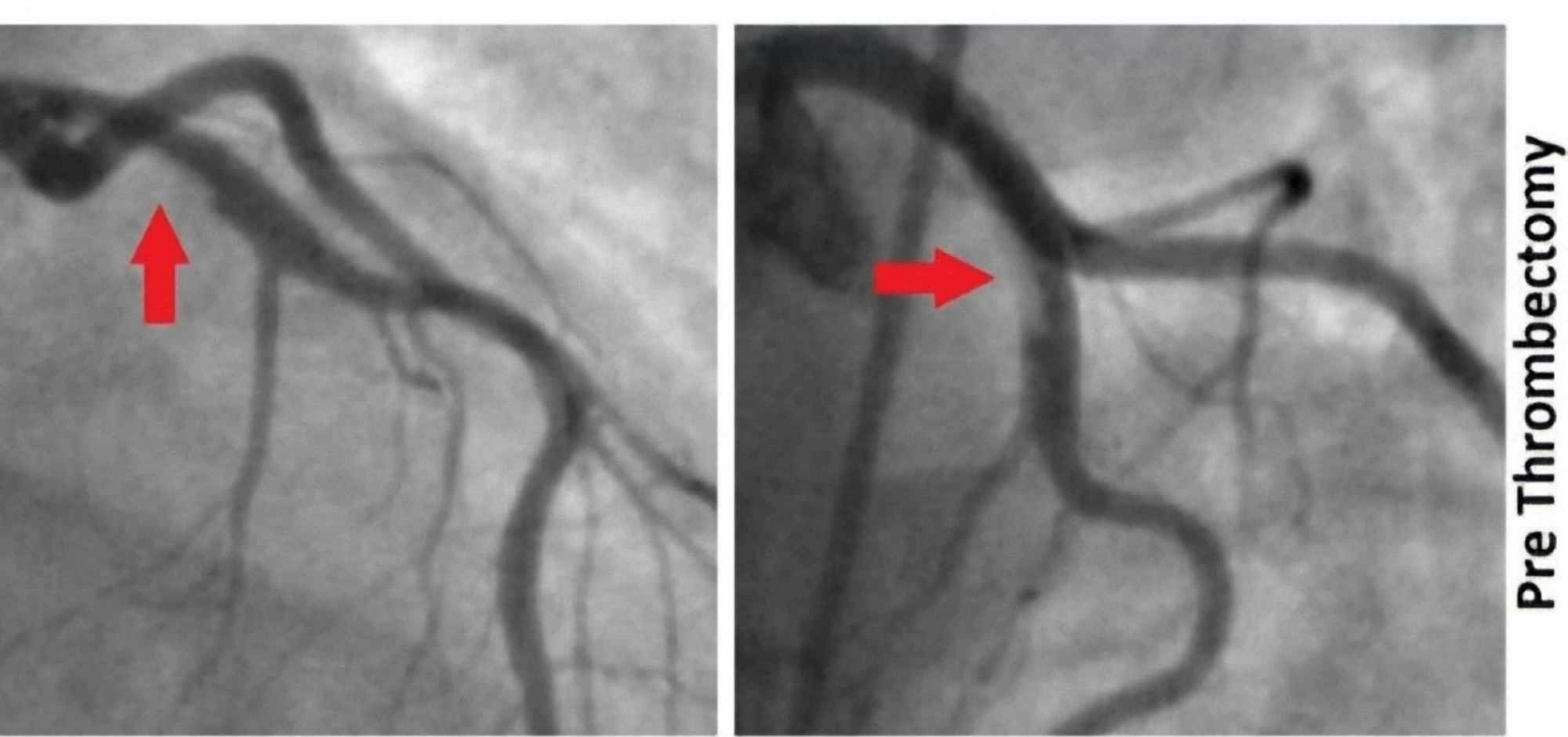
Angiogram of the left coronary artery Thrombus in the left anterior descending artery in the right anterior oblique cranial and the anterio-posterior cranial projections (red arrows)

**Figure 3:**
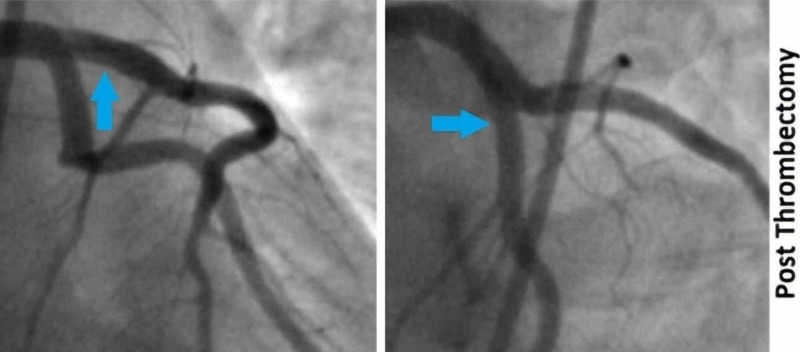
Angiogram of the left coronary artery Resolution of thrombus in the right anterior oblique straight and anterio-posterior cranial projections (blue arrows)

A repeat EKG was done four hours post-presentation, which showed a resolution of ST changes that were seen initially (Figure 1). Troponin was repeated, and this time, it was found to be elevated at 2.3 ng/ml. Despite the atypical nature of chest pain and normal ECHO findings, the dynamic EKG changes and new elevation in troponin prompted a diagnostic coronary angiogram to look for ischemic etiology. Coronary angiogram showed fresh in-situ thrombus in the proximal left anterior descending artery (LAD) with thrombolysis in myocardial infarction (TIMI) 3 flow across the lesion (Figure 2). An aspiration thrombectomy was done, following which there was no residual thrombus burden with TIMI 3 flow (Figure 3). The patient reported relief in her chest pain post-intervention. However, percutaneous intervention with stent placement was not considered appropriate, as there was no evidence of underlying atherosclerotic heart disease.

After stabilizing the patient, further detailed history revealed a previously documented APS with recurrent peripheral arterial thrombosis and multiple peripheral thrombectomies in the past. Prior to her presentation, she was kept on triple anticoagulation maintenance therapy with warfarin, aspirin 81 mg daily, and clopidogrel 75 mg daily for recurrent arterial thrombosis. However, on presentation, her international normalized ratio was in the sub-therapeutic range at 1.1, due to a lack of compliance with her medications, including warfarin.

## Discussion

APS is a multisystem autoimmune disorder characterized by arterial, venous, or small vessel thromboembolic events in the presence of persistent antiphospholipid antibodies [3]. The incidence of arterial thrombosis in these patients has been reported as high as 44% [4]. Moreover, cardiac involvement in APS is not uncommon and occurs chiefly via thrombus formation or direct immunological damage by these circulating antibodies [5]. Valvular involvement is the most common cardiac involvement of APS and can involve up to 38% of patients [6]. Acute myocardial infarction is the presenting manifestation in about 2.8% of patients with APS [7]. Possible mechanisms of myocardial ischemia in patients with APS include accelerated atherosclerosis and coronary thromboembolism. However, APS could potentially be an independent risk factor for accelerated atherosclerosis [8]. Coronary angiography performed in the event of myocardial infarction in APS may demonstrate normal coronaries with or without evidence of acute thrombosis [9], where spontaneous clot lysis can be a possible explanation for this finding.

In our case, coronary angiogram showed acute in-situ thrombosis without evidence of an underlying atherosclerotic coronary artery disease (CAD). The management of acute myocardial infarction in patients with APS is no different than in the general population during the acute phase.

The optimal management of coronary thrombosis without underlying coronary artery disease is unclear, however, an approach of aspiration thrombectomy without stent placement is reasonable [10]. In our patient, there was no evidence of an underlying CAD and hence an approach of aspiration thrombectomy alone was pursued with excellent clinical results. The patient was maintained on long-term anticoagulation with warfarin and clopidogrel as an antiplatelet agent. She remained symptom-free on her two-month follow-up.

## Conclusions

Acute myocardial infarction secondary to in-situ coronary thrombosis is an uncommon yet important presentation found in young patients with APS. It is noteworthy for physicians to be aware of this condition and consider it among the differential diagnosis in patients with a prior diagnosis of APS presenting with chest pain. As most patients with APS are young women, diagnosis of acute myocardial infarction may be easily overlooked, leading to fatal consequences. We emphasize the necessity of considering acute coronary syndrome as a strong possibility in young patients with hyper-coagulable states presenting with chest pain, as a timely diagnosis can be life-saving.
